# Association Between Oral Antihyperglycemic Medications and Erectile Function in Men with Type 2 Diabetes Mellitus

**DOI:** 10.3390/life16040597

**Published:** 2026-04-03

**Authors:** Chia-Hao Wang, Ming-Chieh Lin, Tzu-Jung Fang, Mei-Yueh Lee

**Affiliations:** 1 Division of Endocrinology and Metabolism, Department of Internal Medicine, Kaohsiung Medical University Hospital, Kaohsiung 807377, Taiwan; qoolops2013@gmail.com (C.-H.W.); u102001122@gap.kmu.edu.tw (M.-C.L.); tzujung66@gmail.com (T.-J.F.); 2 Division of Geriatrics and Gerontology, Department of Internal Medicine, Kaohsiung Medical University Hospital, Kaohsiung 807377, Taiwan; 3 School of Medicine, College of Medicine, Kaohsiung Medical University, Kaohsiung 807378, Taiwan; 4 Department of Internal Medicine, Kaohsiung Medical University Gangshan Hospital, Kaohsiung 820111, Taiwan

**Keywords:** erectile dysfunction, type 2 diabetes mellitus, oral antihyperglycemic agents, glycemic control, IIEF-5

## Abstract

**Background/Objectives:** Erectile dysfunction (ED) affects up to 50% of men with type 2 diabetes mellitus (T2DM), yet the independent effects of oral antihyperglycemic medications on erectile function remain controversial. This study investigated associations between commonly prescribed antihyperglycemic medications and erectile function in Taiwanese men with T2DM. **Methods:** This cross-sectional study enrolled 242 Taiwanese men aged 18–80 years with T2DM. Erectile function was assessed using the International Index of Erectile Function–5 (IIEF-5). Participants were categorized by 12-month HbA1c patterns into well-controlled, variably controlled, and poorly controlled groups. Multiple linear regression models adjusted for demographics, metabolic parameters, and comorbidities examined medication–IIEF-5 associations. **Results:** The mean IIEF-5 score was 18.16 ± 5.68. None of the seven oral antihyperglycemic medication classes showed significant independent associations with IIEF-5 scores. However, glycemic control demonstrated a significant association with erectile function (F(2,192) = 3.390, *p* = 0.036), with well-controlled patients showing higher scores than poorly controlled patients (mean difference = 2.488, *p* = 0.032). **Conclusions:** In this cross-sectional study, better glycemic control was associated with improved erectile function in men with T2DM. No significant independent associations were observed between individual oral antihyperglycemic medication classes and erectile function after adjustment for glycemic control and other confounders. These findings suggest that glycemic management, rather than the independent effect of medication class, may be the primary determinant of erectile function in this population; however, causal inferences cannot be drawn from this cross-sectional design.

## 1. Introduction

Erectile dysfunction (ED) represents one of the most common and distressing complications of type 2 diabetes mellitus (T2DM), with prevalence rates ranging from 35% to 75% depending on age and diabetes duration [1,2]. Beyond its impact on sexual health and quality of life, ED in diabetic men serves as an important sentinel marker for systemic vascular disease and cardiovascular risk [1]. The pathophysiology of diabetic ED is multifactorial, involving endothelial dysfunction, autonomic neuropathy, psychological factors, and hormonal alterations [1].

ED has increasingly been recognized as a marker of underlying endocrine and glycemic disorders, often preceding the diagnosis of cardiovascular disease [3]. Specifically, diabetes-induced endothelial dysfunction is characterized by impaired nitric oxide (NO) synthesis and increased oxidative stress, which directly compromise the vasodilatory capacity of penile vasculature [4]. Recent evidence suggests that pyroptosis, a form of inflammatory programmed cell death mediated by inflammasomes, may also contribute to endothelial dysfunction in ED through the release of pro-inflammatory cytokines such as IL-1β and IL-18 [5]. The strong association between diabetes, obesity, and ED underscores the importance of metabolic factors in ED pathogenesis [6,7].

Hyperglycemia and poor glycemic control have been consistently linked to ED development and severity in men with T2DM. However, the role of individual antihyperglycemic medications in either promoting or protecting against ED remains controversial. Recent systematic reviews and meta-analyses have yielded conflicting results [8,9,10]. Some studies suggest that glucagon-like peptide-1 receptor agonists (GLP-1 RAs) may improve erectile function, while others report increased ED risk with metformin or semaglutide [9,10,11,12,13]. A case report documented ED as a possible side effect of metformin therapy, and a recent database study found that prescribing semaglutide for weight loss was associated with increased ED risk [11,13]. Al-Kuraishy et al. reported that diabetic pharmacotherapy may influence testosterone levels and sexual function differently depending on drug class [14]. Thiazolidinediones show potential benefits in some trials, while the effects of SGLT2 inhibitors, DPP-4 inhibitors, and sulfonylureas remain largely unexplored [15]. Sulfonylureas act by stimulating insulin secretion through ATP-sensitive potassium (KATP) channel closure in pancreatic β-cells, and notably, KATP channels are also expressed in penile vascular smooth muscle, where they contribute to vasodilation [6,16,17]. Some studies have suggested potential benefits of GLP-1 receptor agonists when combined with lifestyle modifications and testosterone therapy in diabetic men with hypogonadism, while metformin added to sildenafil may improve outcomes in men with insulin resistance [18,19].

Current clinical guidelines, including the American Diabetes Association (ADA) Standards of Care, emphasize the comprehensive assessment of diabetes-related complications and comorbidities, including those affecting quality of life and sexual health, particularly in patients with cardiovascular risk factors or long-standing disease [20,21]. However, no major guideline has issued specific recommendations regarding the differential effects of individual oral antihyperglycemic agent classes on erectile function, reflecting a notable gap in the current evidence base. A major limitation of existing studies is the inability to disentangle the independent effects of antihyperglycemic medications from glycemic control itself. Because these medications directly influence glycemic status, and glycemic control is a key determinant of erectile function, observed associations may reflect indirect effects mediated through glycemic improvement rather than direct pharmacologic effects. Many prior studies have not adequately addressed this issue, often relying on single cross-sectional HbA1c measurements or failing to account for longitudinal glycemic patterns. Additionally, most existing studies have been conducted in Western populations, with limited data from Asian cohorts, who may exhibit distinct metabolic characteristics and treatment response patterns. The International Index of Erectile Function-5 (IIEF-5), a validated, widely used questionnaire, provides an objective measure of erectile function that enables standardized comparison across studies [13,22].

This study aimed to evaluate the independent associations between seven commonly prescribed oral antihyperglycemic medications and erectile function (primary objective), while also examining the role of glycemic control as a potential mediator (secondary objective). We hypothesized that individual medications would not show significant independent associations with erectile function after comprehensive adjustment for glycemic control and other confounders.

## 2. Materials and Methods

This cross-sectional study was conducted at the out-patient clinic of the Endocrinology and Metabolism Department at Kaohsiung Medical University Hospital, Taiwan, between July 2020 and January 2021. The study protocol was approved by the Institutional Review Board of Kaohsiung Medical University Hospital (KMUHIRB-E(I)-20190350). Informed consent was obtained from all subjects involved in the study.

We enrolled men aged 18–80 years diagnosed with T2DM according to American Diabetes Association criteria, who were followed regularly at our diabetes outpatient clinic [23].

Inclusion criteria were as follows: (1) male gender; (2) age 18–80 years; (3) diagnosed with type 2 diabetes mellitus according to American Diabetes Association (ADA) criteria; (4) receiving outpatient care at Kaohsiung Medical University Hospital; (5) had completed the IIEF-5 questionnaire independently in a private clinical setting; (6) available medication records and laboratory data, including HbA1c values over a 12-month period; and (7) available data on vascular complications (peripheral arterial occlusive disease, coronary artery disease, cerebrovascular accident status).

Exclusion criteria included the following: (1) age <18 or >80 years; (2) type 1 diabetes; (3) severe ED due to anatomical abnormalities or trauma; (4) current use of phosphodiesterase-5 inhibitors; (5) severe psychiatric disorders; (6) acute diabetes complications within 3 months; and (7) incomplete medical records. Of 296 patients screened, 54 were excluded due to not meeting one or more of the above criteria, and 242 were included in the final analysis.

Demographic and clinical data were extracted from electronic medical records at baseline, including age, diabetes duration, body mass index (BMI), and comorbidities. Medication use was documented for all oral antihyperglycemic agents: metformin, SGLT2 inhibitors, DPP-4 inhibitors, thiazolidinediones, sulfonylureas, acarbose, and GLP-1 receptor agonists. Insulin use was also recorded. Medication exposure was defined as current use at the time of IIEF-5 questionnaire completion, recorded as a binary variable without consideration of dose, duration, or combination therapy patterns. Participants were classified as users of a given medication class if the medication was prescribed and actively being taken at the time of questionnaire administration, as documented in the electronic medical records.

Laboratory parameters included fasting plasma glucose, HbA1c, morning fasting serum total testosterone, estimated glomerular filtration rate (eGFR) calculated using the CKD-EPI equation, and urine albumin-to-creatinine ratio (UACR). HbA1c values were collected over a 12-month period preceding the IIEF-5 assessment for glycemic control categorization. All participants had a minimum of two HbA1c measurements available during this period, ensuring that longitudinal glycemic patterns could be assessed for each participant.

Erectile function was evaluated using the Chinese version of the International Index of Erectile Function-5 (IIEF-5) questionnaire, a validated, self-administered tool consisting of five questions assessing erectile function over the past 6 months [13,22]. Each question is scored from 0 to 5 or 1 to 5, yielding a total score range of 1–25. IIEF-5 scores are interpreted as severe ED (1–7), moderate ED (8–11), mild to moderate ED (12–16), mild ED (17–21), and no ED (22–25). The questionnaire was completed independently by participants in a private setting to ensure confidentiality.

Participants were categorized into three glycemic control groups based on longitudinal HbA1c patterns over the 12-month observation period, using the American Diabetes Association glycemic target of HbA1c ≤ 7%: well-controlled (HbA1c ≤ 7% at all clinic visits), variably controlled (HbA1c ≤ 7% at some but not all visits), and poorly controlled (HbA1c > 7% at all visits) [23].

Continuous variables were expressed as mean ± standard deviation (SD) for normally distributed variables, or median (interquartile range) for non-normally distributed variables. Categorical variables were presented as frequencies and percentages. One-way analysis of variance (ANOVA) with the Games–Howell post hoc test was used to compare IIEF-5 scores across glycemic control groups. All statistical analyses were performed using Jamovi version 2.3 (The Jamovi project, Sydney, Australia).

Analysis of covariance (ANCOVA) examined the effect of the glycemic control group on IIEF-5 scores after adjusting for potential confounders: age, diabetes duration, BMI, testosterone, eGFR, and UACR. Covariates were centered at their mean values. Post hoc pairwise comparisons used the Games–Howell method with Tukey adjustment for multiple comparisons.

Multiple linear regression models evaluated associations between individual oral antihyperglycemic medications and IIEF-5 scores. To address potential multicollinearity arising from combination therapy, each medication was examined in a separate fully adjusted regression model rather than entering all medications simultaneously into a single model, controlling for age, diabetes duration, BMI, HbA1c, testosterone, and comorbidities (hypertension, dyslipidemia, cardiovascular disease, and renal disease). Patients receiving multiple drug classes were included as users in each relevant medication model. Bonferroni correction (α = 0.007) was applied for seven medication comparisons. Additionally, subgroup analyses were performed within each glycemic control group (well-controlled, variably controlled, and poorly controlled) to examine whether medication–IIEF-5 associations differed by glycemic status, using separate linear regression models for each group. Two-tailed *p*-values < 0.05 were considered statistically significant, except where Bonferroni correction was applied.

## 3. Results

### 3.1. Baseline Characteristics

Of 296 patients screened, 54 were excluded due to not meeting eligibility criteria, and 242 were included in the final analysis. Participants had a mean age of 50.79 ± 6.99 years (range: 29–60 years) and a mean diabetes duration of 7.58 ± 6.38 years (range: 0–35 years). Mean BMI was 27.95 ± 5.21 kg/m^2^. The overall mean IIEF-5 score was 18.16 ± 5.68 (range: 1–25), indicating mild to moderate erectile dysfunction.

Laboratory parameters showed median testosterone of 356.0 ng/dL (IQR 278.0–459.8), median UACR of 9.0 mg/g, mean eGFR of 86.31 ± 22.79 mL/min/1.73 m^2^, and mean HbA1c of 7.09 ± 1.14%. Regarding comorbidities, hypertension was present in 35.1% and hyperlipidemia in 37.2%. Cardiovascular disease and renal disease were each present in 3.3% of participants, while genitourinary disorders were rare (0.4%). Metformin was most commonly prescribed (72.3%), followed by SGLT2 inhibitors (61.2%), sulfonylureas (48.8%), thiazolidinediones (33.5%), DPP-4 inhibitors (31.7%), acarbose (19.8%), and GLP-1 RAs (5.4%). The detailed baseline characteristics are outlined in Table 1.

Baseline characteristics across the three glycemic control groups are presented in Supplementary Table S1. The three groups were well-balanced across most clinical and laboratory parameters. A significant difference was observed only for diabetes duration (F = 3.595, *p* = 0.030) and total OAD types (F = 30.693, *p* < 0.001), with the poorly controlled group having longer diabetes duration and taking more medications. Medication distribution also differed significantly across groups for several OHA classes. Further supporting the presence of confounding by indication, HbA1c levels were significantly higher among users of sulfonylureas, thiazolidinediones, and GLP-1 receptor agonists compared with non-users, consistent with preferential prescribing to patients with poorer glycemic control (Supplementary Table S2).

### 3.2. Primary Outcome: Associations Between Oral Antihyperglycemic Medications and Erectile Function

**Figure 1:**
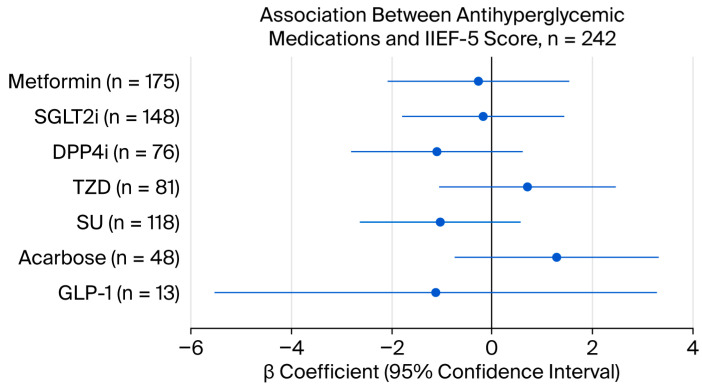
Association between oral antihyperglycemic medications and IIEF-5 scores in men with type 2 diabetes mellitus (n = 242). β represents the difference in IIEF-5 score between medication users and non-users; positive values indicate better erectile function in users. The vertical reference line at β = 0 indicates no association. Models were adjusted for age, diabetes duration, BMI, HbA1c, testosterone, ACR, eGFR, and comorbidities (hypertension, dyslipidemia, cardiovascular disease, and renal disease). Bonferroni-corrected significance threshold: α = 0.007.

Table 2 and Figure 1 present associations between individual medications and IIEF-5 scores. After covariate adjustment, no medication class demonstrated a statistically significant association with IIEF-5 scores (all *p* > 0.05). Even after Bonferroni correction (α = 0.007), significance was not achieved for any medication. The wide confidence interval observed for GLP-1 receptor agonists reflects the limited sample size for this subgroup (n = 13). Detailed regression coefficients and confidence intervals are presented in Table 2.

### 3.3. Secondary Outcome: Association Between Glycemic Control and Erectile Function

ANCOVA revealed a significant association between glycemic control and IIEF-5 scores (F(2,192) = 3.390, *p* = 0.036). A graded relationship was observed, with well-controlled patients having higher scores than poorly controlled patients.

**Figure 2:**
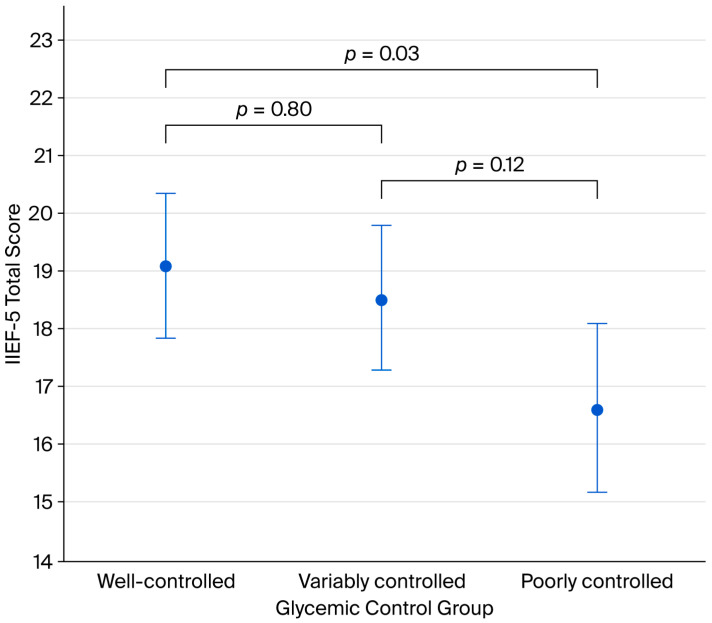
Comparisons of IIEF-5 scores between glycemic control groups.

Post hoc analysis showed a significant difference between well-controlled and poorly controlled groups (mean difference = 2.488, *p* = 0.032), while differences between well-controlled and variably controlled groups and between variably controlled and poorly controlled groups did not reach significance. These results are illustrated in Table 3, Table 4 and Figure 2.

**Table 3:** Analysis of covariance for IIEF-5 Score by glycemic control group.

Source	Sum of Squares	df	Mean Square	F	*p*-Value
Glycemic control group	195.64	2	97.82	3.39	0.036
Age (years)	158.91	1	158.91	5.50	0.020
Diabetes duration (years)	120.45	1	120.45	4.17	0.042
Body mass index (kg/m^2^)	2.14	1	2.14	0.07	0.786
eGFR (mL/min/1.73 m^2^)	38.73	1	38.73	1.34	0.248
ACR (mg/g)	66.16	1	66.16	2.29	0.132
Testosterone (ng/dL)	2.08	1	2.08	0.07	0.789
Residual	5539.5	192	28.85		

Dependent variable: International Index of Erectile Function-5 (IIEF-5) total score. Covariates were centered at their mean values. Glycemic control groups: well-controlled (HbA1c ≤ 7% at all visits), variably controlled (HbA1c ≤ 7% at some visits), poorly controlled (HbA1c > 7% at all visits). Abbreviations: eGFR, estimated glomerular filtration rate; ACR, albumin-to-creatinine ratio.

**Table 4:** Subgroup analysis by glycemic control status.

Subgroup	OHA Class	β (Beta)	95% CI Lower	95% CI Upper	*p*-Value	n (Users)
Group A (Well-Controlled, n = 85)	Metformin	2.19	−1.64	6.02	0.268	54
	SGLT2 inhibitors	0.87	−2.05	3.78	0.562	47
	DPP-4 inhibitors	−2.49	−5.46	0.48	0.106	36
	Thiazolidinediones	0.7	−2.8	4.19	0.698	20
	Sulfonylureas	−1.72	−5.03	1.58	0.312	27
	Acarbose	2.28	−1.7	6.26	0.266	20
Group B (Variably Controlled, n = 93)	Metformin	−2.94	−6.31	0.42	0.092	76
	SGLT2 inhibitors	−0.88	−3.45	1.69	0.504	57
	DPP-4 inhibitors	−0.34	−3.55	2.87	0.837	18
	Thiazolidinediones	1.67	−0.99	4.34	0.224	30
	Sulfonylureas	−0.15	−2.52	2.22	0.901	51
	Acarbose	2.35	−1.83	6.52	0.275	9
	GLP-1 RA	0.61	−5.54	6.77	0.846	7
Group C (Poorly Controlled, n = 64)	Metformin	−2.91	−6.4	0.58	0.112	45
	SGLT2 inhibitors	−1.32	−5.02	2.37	0.488	44
	DPP-4 inhibitors	0.25	−3.33	3.84	0.891	22
	Thiazolidinediones	0.47	−3.03	3.98	0.792	31
	Sulfonylureas	−2.23	−5.52	1.06	0.193	40
	Acarbose	0.59	−3.33	4.51	0.768	19
	GLP-1 RA	−3.54	−10.84	3.75	0.347	5

Notes: β = unstandardized regression coefficient (ANCOVA), adjusted for age, diabetes duration, BMI, HbA1c, testosterone, ACR, eGFR, and comorbidities (hypertension, dyslipidemia, cardiovascular disease, and renal disease). Outcome: IIEF-5 total score. All *p*-values > 0.05 (non-significant). 95% CI = 95% confidence interval. Group A: well-controlled (HbA1c ≤ 7% at all clinic visits, n = 85); Group B: variably controlled (HbA1c ≤ 7% at some but not all visits, n = 93); Group C: poorly controlled (HbA1c > 7% at all clinic visits, n = 64). GLP-1 RA excluded from Group A (n = 1, insufficient sample size). Analyses performed in Jamovi 2.3.

### 3.4. Secondary Outcome: Associations Between Demographic and Metabolic Parameters and Erectile Function

Among covariates, age (*p* = 0.02) and diabetes duration (*p* = 0.042) were significantly associated with IIEF-5 scores, while BMI, eGFR, UACR, and testosterone were not (all *p* > 0.05). Pearson correlation analysis confirmed no significant association between serum testosterone levels and IIEF-5 scores (r = 0.038, 95% CI: −0.092 to 0.167, *p* = 0.562).

### 3.5. Subgroup Analysis: Medication Associations Within Glycemic Control Groups

**Figure 3:**
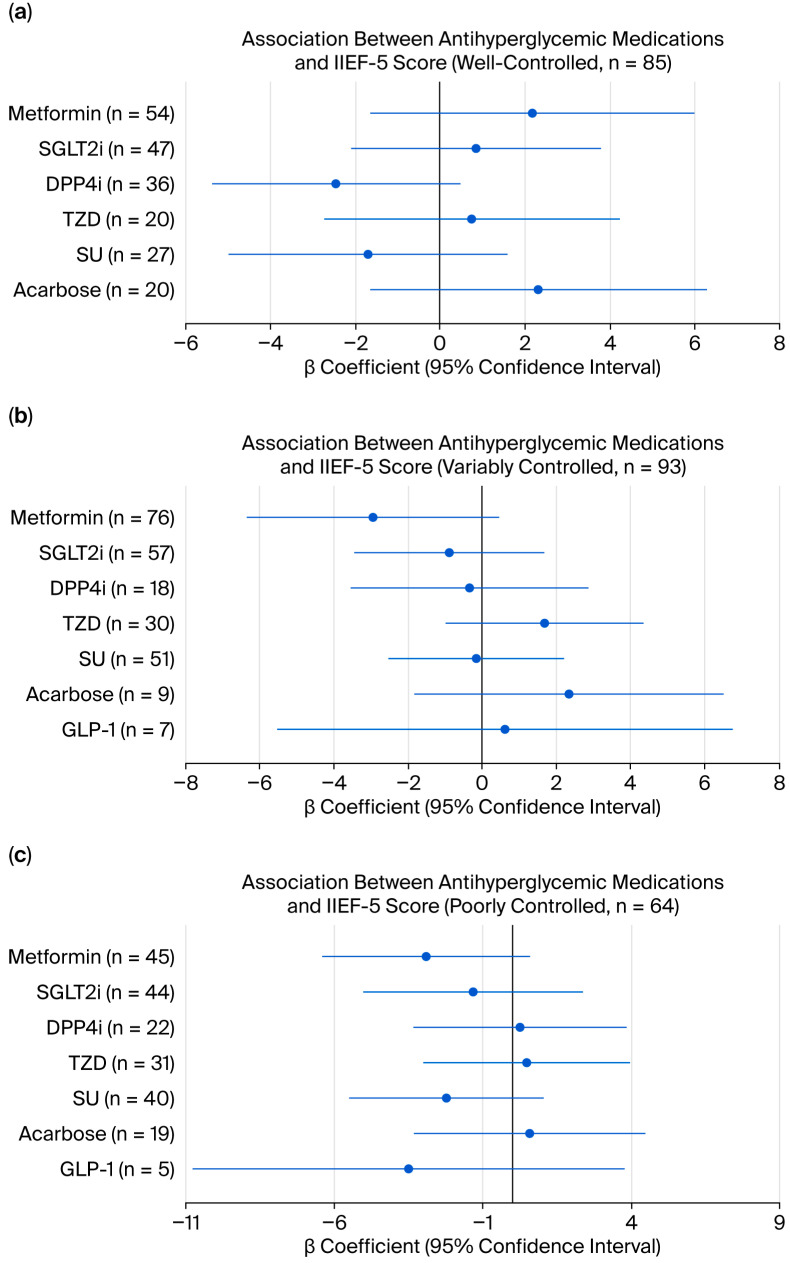
Forest plots showing adjusted regression coefficients (β) and 95% confidence intervals for associations between oral antihyperglycemic medications and IIEF-5 scores within glycemic control subgroups: (**a**) well-controlled group (n = 85); (**b**) variably controlled group (n = 93); (**c**) poorly controlled group (n = 64). β represents the difference in IIEF-5 score between medication users and non-users; positive values indicate better erectile function in users. The vertical reference line at β = 0 indicates no association. Models were adjusted for age, diabetes duration, BMI, HbA1c, testosterone, ACR, eGFR, and comorbidities (hypertension, dyslipidemia, cardiovascular disease, and renal disease).

Subgroup analyses within each glycemic control group (well-controlled, n = 85; variably controlled, n = 93; poorly controlled, n = 64) revealed no significant medication–IIEF-5 associations in any group (all *p* > 0.05). GLP-1 receptor agonists were excluded from the well-controlled group analysis due to insufficient sample size (n = 1). These consistent null findings across all three glycemic control subgroups reinforce the primary analysis results. A visual summary of these subgroup analyses is provided in Figure 3.

## 4. Discussion

### 4.1. Principal Findings

This cross-sectional study’s primary finding was that none of the seven commonly prescribed oral antihyperglycemic medications demonstrated significant independent associations with erectile function in 242 Taiwanese men with T2DM after comprehensive adjustment for confounders. This null finding across all medication classes suggests that previously reported associations may have been confounded by glycemic control or other metabolic factors. Secondarily, we found that glycemic control status showed a significant graded relationship with erectile function, with well-controlled patients scoring 2.488 points higher than poorly controlled patients (*p* = 0.032). Together, these findings suggest that the effect of antihyperglycemic medications on erectile function may operate largely through improvements in glycemic control, rather than through direct pharmacological mechanisms. However, longitudinal studies with mediation analyses are needed to confirm this hypothesis.

### 4.2. Glycemic Control and Erectile Function

The significant association between glycemic control and erectile function, with a graded relationship across three glycemic control groups, corroborates extensive literature linking hyperglycemia to ED pathophysiology [1,2]. This finding was further supported by a significant negative correlation between 12-month mean HbA1c and IIEF-5 scores (r = −0.190, *p* = 0.006). This clinically meaningful pattern suggests that achieving optimal glycemic control should remain a primary therapeutic goal for ED prevention in T2DM patients.

### 4.3. Comparison with Previous Studies

Our finding that no individual medications showed significant associations contrasts with some previous studies. A Mendelian randomization study reported that metformin increased ED risk, while a meta-analysis suggested that GLP-1 RAs may improve erectile function [9,10,12]. However, these discrepancies likely reflect methodological differences. Many previous studies lacked adequate adjustment for glycemic control when evaluating medication effects. For instance, while Giagulli et al. reported that adding liraglutide to lifestyle changes, metformin, and testosterone therapy improved erectile function in diabetic obese men with hypogonadism, and Rey-Valzacchi et al. found that metformin addition to sildenafil improved outcomes in insulin-resistant men, these studies did not fully account for the glycemic improvements that accompanied medication use [18,19]. Similarly, Defeudis et al. examined the role of antihyperglycemic drugs and diet on erectile function in a population with prediabetes and diabetes, highlighting the complex interplay between metabolic control and medication effects [24]. An exploratory randomized controlled trial on continuous subcutaneous insulin infusion also demonstrated potential benefits for ED in T2DM patients, further supporting the primacy of glycemic control [25].

Our study addressed this through a dual approach: (1) controlling for baseline HbA1c in medication regression models to isolate medication-specific effects from concurrent glycemic status; and (2) examining longitudinal glycemic control patterns (sustained good control, fluctuating control, or sustained poor control) to assess the impact of long-term metabolic status beyond what single time-point HbA1c measurements can capture. Our subgroup analysis further supports this conclusion. When medication associations were examined separately within each glycemic control group (well-controlled, variably controlled, and poorly controlled), the null findings persisted consistently across all subgroups. This consistency across varying glycemic environments suggests that the lack of medication-specific effects is unlikely to be fully explained by confounding from glycemic control.

### 4.4. Other Risk Factors

The independent associations of age (*p* = 0.020) and diabetes duration (*p* = 0.042) align with established ED risk factors [1,2]. However, when HbA1c was additionally controlled in medication regression models, these associations were attenuated, suggesting that the effects of age and diabetes duration on erectile function may be partially mediated through glycemic control pathways. Interestingly, testosterone showed no significant association (*p* = 0.789), contrasting with some findings in the literature. This may reflect our population’s relatively preserved testosterone levels (median 356 ng/dL) or a predominance of vascular mechanisms over hormonal factors.

### 4.5. Clinical Implications

From a clinical perspective, our findings provide reassurance that commonly prescribed oral antidiabetic medications do not independently worsen erectile function when glycemic control is appropriately managed. This is particularly important for patient counseling, as concerns about medication side effects can impact treatment adherence. Clinicians should emphasize that achieving optimal glycemic control—regardless of specific medication regimen—represents the most evidence-based strategy for ED prevention.

### 4.6. Mechanistic Considerations

The mechanistic pathways linking glycemic control to erectile function involve multiple systems [24,26]. Chronic hyperglycemia promotes endothelial dysfunction through increased oxidative stress, advanced glycation end products, and impaired nitric oxide bioavailability—all critical for penile vascular health. Additionally, hyperglycemia accelerates peripheral neuropathy and microvascular complications. Our findings support the hypothesis that medication benefits occur primarily through these glycemic-mediated pathways. At the cellular level, hyperglycemia-induced endothelial dysfunction involves multiple mechanisms including reduced NO bioavailability, increased production of reactive oxygen species (ROS), and activation of inflammatory pathways [4]. Emerging evidence indicates that pyroptosis-mediated endothelial cell death may represent a key mechanism linking chronic inflammation to ED development [5]. Additionally, ATP-sensitive potassium (KATP) channels play an important role in penile vascular smooth muscle relaxation, and their function may be impaired in diabetic states [17,27].

### 4.7. Strengths and Limitations

Several limitations warrant consideration. First, the cross-sectional design precludes causal inference, as medication exposure and erectile function were assessed simultaneously. Prospective longitudinal studies are needed to establish directionality. Second, the small number of GLP-1 receptor agonist users (n = 13, 5.4%) substantially limited statistical power for this subgroup, and the study was underpowered to detect small-to-moderate effect sizes for this medication class. The null finding for GLP-1 receptor agonists should therefore be interpreted with caution, and larger prospective studies are needed to clarify its potential effects on erectile function. Third, findings from our Taiwanese cohort may not be fully generalizable to other ethnic groups. Fourth, the actual age distribution of our cohort (29–60 years) reflects the real-world demographic profile of men attending our diabetes outpatient clinic who met all eligibility criteria. Older patients may have been less represented due to several factors: higher rates of incomplete medical records; higher prevalence of current phosphodiesterase-5 inhibitor use; and occurrence of acute diabetes complications within the preceding 3 months (with both of the latter being exclusion criteria). These factors are all more prevalent in older patients with long-standing T2DM. This may limit generalizability to older men with T2DM, in whom ED prevalence is substantially higher. Fifth, several potential confounders were not captured, including smoking status, alcohol consumption, physical activity, and psychological factors such as depression and anxiety, which may have contributed to residual confounding. Sixth, medication exposure was defined as current use at the time of questionnaire completion, without information on treatment duration, dosage, or combination therapy patterns, which may not fully capture cumulative pharmacological exposure. Furthermore, as patients may concurrently receive multiple drug classes, overlapping medication use may introduce residual confounding between treatment groups, despite the use of separate regression models for each medication class. Taken together, these limitations preclude definitive conclusions regarding direct pharmacological effects of individual OHA classes on erectile function.

Strengths include comprehensive adjustment for multiple confounders, including baseline HbA1c in medication models and longitudinal glycemic control patterns in secondary analysis, validated IIEF-5 assessment, clinically relevant categorization reflecting real-world patterns, and evaluation of seven medication classes simultaneously. The use of 12-month glycemic control patterns (rather than a single time-point HbA1c) in our secondary analysis provides a more robust characterization of sustained metabolic status.

## 5. Conclusions

In conclusion, this cross-sectional study found that better glycemic control was associated with improved erectile function in men with T2DM, following a graded relationship. No individual oral antihyperglycemic medication classes were significantly associated with erectile function after covariate adjustment, while age and diabetes duration remained important non-glycemic factors. These findings suggest that any potential effects of medications on erectile function may be mediated through glycemic control rather than direct pharmacological mechanisms. However, given the cross-sectional design, these results should be interpreted as the absence of a detected association rather than definitive evidence of no effect. Further prospective studies are needed to clarify the independent effects of individual medication classes on erectile function.

## Figures and Tables

**Table 1:** Baseline characteristics of study participants (n = 242).

Characteristic	Value	Range
Demographics		
Age (years)	50.79 ± 6.99	29–60
Diabetes duration (years)	7.58 ± 6.38	0–35
Clinical characteristics		
Body mass index (kg/m^2^)	27.95 ± 5.21	17.6–53.4
IIEF-5 score	18.16 ± 5.68	1–25
Laboratory parameters		
Testosterone (ng/dL)	356 (278–459.8)	53–1025.5
Albumin-to-creatinine ratio (mg/g)	9 (4.5–30.8)	1.10–5052
eGFR (mL/min/1.73 m^2^)	86.31 ± 22.79	17.3–151.3
HbA1c (%)	7.09 ± 1.14	5.30–12.20
Comorbidities		
Hypertension	85 (35.1%)	—
Hyperlipidemia	90 (37.1%)	—
Hypogonadism	0 (0.0%)	—
Genitourinary disorders	1 (0.4%)	—
Diabetic complications		
Cardiovascular disease	8 (3.3%)	—
Renal disease	8 (3.3%)	—
Oral antihyperglycemic agents		
Insulin therapy		
Basal insulin	29 (11.9%)	—
Basal-bolus or mixed insulin	22 (9.1%)	—
Any insulin use	51 (21.1%)	—
Metformin	175 (72.3%)	—
SGLT2 inhibitors	148 (61.2%)	—
DPP-4 inhibitors	76 (31.4%)	—
Thiazolidinediones	81 (33.5%)	—
Sulfonylureas	118 (48.8%)	—
Acarbose	48 (19.8%)	—
GLP-1 receptor agonists	13 (5.4%)	—

Data are presented as means ± standard deviation for normally distributed continuous variables, medians for skewed variables (standard deviation > mean), or n (%) for categorical variables. For skewed variables (testosterone, albumin-to-creatinine ratio), the median is shown instead of the mean. Range represents minimum–maximum values. Abbreviations: IIEF-5, International Index of Erectile Function-5 (score range 5–25; higher scores indicate better erectile function); eGFR, estimated glomerular filtration rate calculated by CKD-EPI equation; HbA1c, glycated hemoglobin; SGLT2, sodium–glucose cotransporter-2; DPP-4, dipeptidyl peptidase-4; GLP-1, glucagon-like peptide-1.

**Table 2:** Associations between oral antihyperglycemic agents and IIEF-5 scores (fully adjusted model).

Medication	n (Users, % of Total)	β (95% CI)	*p*-Value
Metformin	175 (72.3%)	−0.27 (−2.09, 1.54)	0.770
SGLT2 inhibitors	148 (61.2%)	−0.18 (−1.79, 1.44)	0.830
DPP-4 inhibitors	76 (31.4%)	−1.10 (−2.81, 0.62)	0.212
Thiazolidinediones	81 (33.5%)	0.71 (−1.05, 2.47)	0.431
Sulfonylureas	118 (48.8%)	−1.03 (−2.63, 0.56)	0.207
Acarbose	48 (19.8%)	1.28 (−0.75, 3.32)	0.219
GLP-1 receptor agonists	13 (5.4%)	−1.12 (−5.53, 3.28)	0.618

Fully Adjusted Model: Adjusted for age, diabetes duration, BMI, HbA1c, testosterone, ACR, eGFR, and comorbidities (hypertension, dyslipidemia, cardiovascular disease, and renal disease). β = Regression coefficient representing the difference in IIEF-5 score between medication users and non-users. CI = Confidence interval. A positive β indicates better erectile function in users; a negative β indicates worse. Using Bonferroni correction for multiple testing (7 comparisons), the adjusted significance threshold would be *p* < 0.007. Abbreviations: IIEF-5, International Index of Erectile Function-5; SGLT2, sodium-glucose cotransporter-2; DPP-4, dipeptidyl peptidase-4; GLP-1, glucagon-like peptide-1.

## Data Availability

The data presented in the study are available on request from the corresponding author. The data are not publicly available due to privacy restrictions.

## Supplementary Materials

The following supporting information can be downloaded at: https://www.mdpi.com/article/10.3390/life16040597/s1. Table S1: Baseline Characteristics by Glycemic Control Group (n = 242); Table S2: HbA1c Comparison Between Users and Non-Users of Each Antihyperglycemic Medication.

## Abbreviations

The following abbreviations are used in this manuscript:

ED	Erectile dysfunction
T2DM	Type 2 diabetes mellitus
IIEF-5	International Index of Erectile Function–5
GLP-1 RA	Glucagon-like peptide-1 receptor agonist
ADA	American Diabetes Association
BMI	Body mass index
eGFR	Estimated glomerular filtration rate
UACR	Urine albumin-to-creatinine ratio
SD	Standard deviation
ANOVA	One-way analysis of variance
ANCOVA	Analysis of covariance
CI	Confidence interval
SGLT2	Sodium–glucose cotransporter-2
DPP-4	Dipeptidyl peptidase-4
IQR	Interquartile Range
