# Supplementary material for: Association Between Oral Antihyperglycemic Medications and Erectile Function in Men with Type 2 Diabetes Mellitus
Source: Life (Basel). 2026 Apr 3;16(4):597. doi: 10.3390/life16040597 (PMC13117611; doi:10.3390/life16040597)
Supplement: Supplementary file 1 [file life-16-00597-s001.zip › life-4157598-supplementary.pdf]

Supplementary Table S1. Baseline Characteristics by Glycemic Control Group (N = 242)

Characteristic	Well-controlled (A, n = 85)	Variably controlled (B, n = 93)	Poorly controlled (C, n = 64)	Statistic	p-value
**Continuous variables**					
**Age (years), mean ± SD**	50.68 ± 6.90	51.14 ± 7.18	50.41 ± 6.93	F = 0.218	0.805
**BMI (kg/m²), mean ± SD**	27.61 ± 4.36	27.95 ± 5.33	28.39 ± 6.07	F = 0.364	0.695
**Diabetes duration (years), mean ± SD**	6.51 ± 5.40	7.34 ± 6.51	9.36 ± 7.09	F = 3.595	0.030*
**HbA1c (%), mean ± SD**	6.29 ± 0.42	6.91 ± 0.69	8.42 ± 1.16	F = 111.419	< 0.001*
**ACR (mg/g), median**	7.80	7.50	10.50	F = 0.764	0.468
**eGFR (mL/min/1.73m²), mean ± SD**	87.61 ± 21.57	85.07 ± 23.63	86.37 ± 23.36	F = 0.279	0.757
**Total OAD types, mean ± SD**	2.47 ± 1.02	2.84 ± 1.14	3.69 ± 0.91	F = 30.693	< 0.001*
**Comorbidities, n (%)**					
**Hypertension**	30 (40.5%)	32 (42.1%)	23 (43.4%)	$\chi^2 = 0.106$	0.948
**Hyperlipidemia**	34 (45.9%)	34 (44.7%)	22 (41.5%)	$\chi^2 = 0.254$	0.881
**Cardiovascular disease**	1 (1.4%)	6 (7.9%)	1 (1.9%)	$\chi^2 = 5.04$	0.080
**Antihyperglycemic medications, n (%)**					
**Metformin**	54 (63.5%)	76 (81.7%)	45 (70.3%)	$\chi^2 = 8.24$	0.016*
**SGLT2 inhibitors**	47 (55.3%)	57 (61.3%)	44 (68.8%)	$\chi^2 = 2.78$	0.249
**DPP-4 inhibitors**	36 (42.4%)	18 (19.4%)	22 (34.4%)	$\chi^2 = 10.6$	0.005*
**Thiazolidinediones**	20 (23.5%)	30 (32.3%)	31 (48.4%)	$\chi^2 = 10.3$	0.006*
**Sulfonylureas**	27 (31.8%)	51 (54.8%)	40 (62.5%)	$\chi^2 = 16.0$	< 0.001*
**Acarbose**	20 (23.5%)	9 (9.7%)	19 (29.7%)	$\chi^2 = 10.7$	0.005*
**GLP-1 receptor agonists**	1 (1.2%)	7 (7.5%)	5 (7.8%)	$\chi^2 = 4.54$	0.103

*Data are presented as mean ± SD for normally distributed continuous variables, median for skewed variables (ACR), or n (%) for categorical variables. One-way ANOVA (Welch's) was used for continuous variables; chi-square test for categorical variables. * $p < 0.05$. Abbreviations: BMI, body mass index; HbA1c, glycated haemoglobin; ACR, albumin-to-creatinine ratio; eGFR, estimated glomerular filtration rate; OAD, oral antidiabetic drug; SGLT2, sodium-glucose cotransporter-2; DPP-4, dipeptidyl peptidase-4; GLP-1, glucagon-like peptide-1.*

Supplementary Table S2. HbA1c Comparison Between Users and Non-Users of Each Antihyperglycemic Medication

Medication	HbA1c, Users (mean ± SD)	HbA1c, Non-users (mean ± SD)	p-value	N (Users / Non-users)
**Metformin**	7.16 ± 1.18	6.93 ± 1.05	0.184	175 / 65
**SGLT2i**	7.11 ± 1.02	7.06 ± 1.32	0.704	148 / 94
**DPP-4i**	6.99 ± 1.22	7.15 ± 1.11	0.331	76 / 164
**TZD**	7.41 ± 1.41	6.93 ± 0.95	0.002[a]	81 / 161
**Sulfonylurea**	7.37 ± 1.20	6.83 ± 1.02	< .001	118 / 124
**Acarbose**	7.21 ± 1.01	7.06 ± 1.17	0.418	48 / 194
**GLP-1 RA**	8.15 ± 1.48	7.03 ± 1.10	< .001[a]	13 / 229

Data are presented as mean ± SD. p-values from independent samples t-test. Significant p-values ($p < 0.05$) are shown in red. [a] Levene's test significant ($p < .05$); equal variances not assumed.
